# Fabrication of Flexible All-Solid-State Asymmetric Supercapacitor Device via Full Recycling of Heated Tobacco Waste Assisted by PLA Gelation Template Method

**DOI:** 10.3390/gels9020097

**Published:** 2023-01-23

**Authors:** Suk Jekal, Min-Sang Kim, Dong-Hyun Kim, Jungchul Noh, Ha-Yeong Kim, Jiwon Kim, Hyeonseok Yi, Won-Chun Oh, Chang-Min Yoon

**Affiliations:** 1 Department of Chemical and Biological Engineering, Hanbat National University, Daejeon 34158, Republic of Korea; 2 McKetta Department of Chemical Engineering and Texas Material Institute, The University of Texas at Austin, Austin, TX 78712, USA; 3 Institute for Materials Chemistry and Engineering, Kyushu University, Fukuoka 816-8580, Japan; 4 Department of Advanced Materials Science & Engineering, Hanseo University, Seosan-si 31962, Republic of Korea

**Keywords:** flexible supercapacitor, heated tobacco, PLA gelation, asymmetric supercapacitor, recycling

## Abstract

In this study, a flexible all-solid-state asymmetric supercapacitor (FASC) device has been successfully fabricated via full recycling of heated tobacco waste (HTW). Tobacco leaves and cellulose acetate tubes have been successfully carbonized (HTW-C) and mixed with metal oxides (MnO_2_ and Fe_3_O_4_) to obtain highly active materials for supercapacitors. Moreover, poly(lactic acid) (PLA) filters have been successfully dissolved in an organic solvent and mixed with the as-prepared active materials using a simple paste mixing method. In addition, flexible MnO_2_- and Fe_3_O_4_-mixed HTW-C/PLA electrodes (C-MnO_2_/PLA and C-Fe_3_O_4_/PLA) have been successfully fabricated using the drop-casting method. The as-synthesized flexible C-MnO_2_/PLA and C-Fe_3_O_4_/PLA electrodes have exhibited excellent electrical conductivity of 378 and 660 μS cm^−1^, and high specific capacitance of 34.8 and 47.9 mF cm^−2^ at 1 mA cm^−2^, respectively. A practical FASC device (C-MnO_2_/PLA//C-Fe_3_O_4_/PLA) has been assembled by employing the C-MnO_2_/PLA as the positive electrode and C-Fe_3_O_4_/PLA as the negative electrode. The as-prepared FASC device showed a remarkable capacitance of 5.80 mF cm^−2^ at 1 mA cm^−2^. Additionally, the FASC device manifests stable electrochemical performance under harsh bending conditions, verifying the superb flexibility and sustainability of the device. To the best of our knowledge, this is the first study to report complete recycling of heated tobacco waste to prepare the practical FASC devices. With excellent electrochemical performance, the experiments described in this study successfully demonstrate the possibility of recycling new types of biomass in the future.

## 1. Introduction

As the technology improves, the energy consumption is increasing rapidly over the years, which is also causing the depletion of energy and environmental problems [1,2]. With the increase in energy demand, inevitably, more wastes and biomasses are generated to fulfill the standard and convenience of human being [3,4]. To solve both the energy crisis and biomass generation, recent studies are focusing on the recycling of various biomass wastes into materials for various energy storage devices [5,6,7]. Among them, supercapacitors are receiving wide attention owing to their various positive characteristics, including easy applicability of biomass into active material, fast charging-discharging rate, long-term stability, and excellent power density [8,9,10,11,12,13,14]. Supercapacitors can be classified into three types: electric double-layer capacitors (EDLC), pseudocapacitors, and hybrid capacitors. Each supercapacitor is prepared with different active materials on the electrodes, and thus, different charging-discharging mechanisms [15,16,17]. In EDLC, carbon materials such as activated carbon, carbon nanotubes (CNT), carbon nanofibers (CNF), and graphene are employed as active materials, and electrical charges are physically stored on the surface of the active materials [18,19]. Therefore, the capacitance of EDLC is significantly affected by the electrical conductivity and surface area of the carbon materials [20,21,22]. Moreover, pseudocapacitors charge and discharge electrons via electrochemical or redox reactions. In this regard, typical active materials employed in pseudocapacitors are metal oxides (MnO_2_ and RuO_2_) and their related materials, including MnO_2_-nanowhiskers [23,24,25]. In addition, conducting polymers like polypyrrole, polyaniline, polyimide, and polydopamine are used as materials for pseudocapacitors [26,27]. In general, pseudocapacitors are fabricated with higher energy densities than EDLC, owing to the presence of redox reactions.

To incorporate the advantages of EDLC and pseudocapacitors, various types of hybrid capacitors have been introduced. For instance, Noh et al. have reported a high-performance hybrid capacitor composed of highly conductive carbon fibers composited with manganese oxide (MnO_2_) and molybdenum oxide (MoO_3_), which successfully demonstrates the positive aspects of both EDLC and pseudocapacitors [28]. Also, MXenes, two-dimensional (2D) transition metal carbides and nitrides, are studied as an electrochemically stable material for supercapacitor application [29]. Moreover, such hybrid supercapacitors are fabricated as all-solid-state supercapacitor devices with flexible characteristics by employing flexible carbon substrates with the addition of pseudo-materials and the final construction of devices with solid electrolytes using polyvinyl alcohol (PVA) [30,31,32]. For example, Li et al. have successfully fabricated a flexible asymmetric supercapacitor (ASC) employing CoAl-layered double hydroxide (CoAl-LDH)/carbon cloth (CC) and reduced graphene oxide (rGO)/CC electrodes, which exhibit excellent electrochemical performance and durability under various bending conditions [33]. Also, Liu et al. have combined a MnO_2_-coated graphene foam/carbon nanotube hybrid film and a PPy-coated graphene foam/carbon nanotube hybrid film to assemble an ASC, demonstrating great potential as a hybrid and flexible supercapacitor device [34].

With the advent of Industry 4.0, eco-friendly and biomass recycling methods have received wide attention. There are many ways to recycle biomass, but one of the most promising methods is to simply collect the biomass and carbonize it for energy storage device applications. However, the grade of the biomass or carbon precursors is important for obtaining excellent carbon quality. Another important factor of biomass-derived carbon is its abundance and ease of accessibility. One biomass that perfectly matches such requirements is tobacco waste, as 5.7 trillion tobacco butts are generated annually and thrown away [35,36]. Thereby, some successful carbonization methods of tobacco waste including filters for supercapacitor applications have been reported [37,38,39]. Recently, heated tobacco products have been developed, which have different smoking mechanisms than conventional tobacco. Specifically, conventional tobacco wastes are only left with filter parts, but heated tobacco wastes are left with almost all wastes owing to the steaming and smoking mechanism. In this regard, heated tobacco waste could be a major pollution factor in the future.

In this study, we introduce a facile route for constructing a flexible all-solid-state asymmetric supercapacitor (FASC) device via full recycling of heated tobacco waste assisted by the poly(lactic acid) (PLA) gelation template method. To the best of our knowledge, this is the first study to report a fabrication method for a FASC device by full recycling of heated tobacco waste. Heated tobacco waste has been collected and divided into tobacco leaves, filter tubes, and PLA filters. Tobacco leaves and filter tubes are employed as carbon precursors, and PLA filters are used as flexible binding substrates. Heated tobacco waste-derived carbon (HTW-C) materials are mixed with metal oxides (MnO_2_ and Fe_3_O_4_) to attain both EDLC and pseudocapacitance. Using a simple paste mixing and drop-casting method, flexible electrodes of MnO_2_-mixed HTW-C/PLA (C-MnO_2_/PLA) and Fe_3_O_4_-mixed HTW-C/PLA (C-Fe_3_O_4_/PLA) have successfully been synthesized. Each flexible electrode manifests excellent electrical conductivity and capacitance of 378 μS cm^−1^ and 34.8 mF cm^−2^ for C-MnO_2_/PLA, and 660 μS cm^−1^ and 47.9 mF cm^−2^ for C-Fe_3_O_4_/PLA. Moreover, the practical FASC device has successfully been assembled using two electrodes (C-MnO_2_/PLA//C-Fe_3_O_4_/PLA), which displays an excellent capacitance of 5.80 mF cm^−2^ at 1 mA cm^−2^ with an extended voltage of 2.0 V. Also, the as-prepared C-MnO_2_/PLA//C-Fe_3_O_4_/PLA FASC device has exhibited stable electrochemical performance under various harsh bending conditions of 45, 90, and 180°. Owing to the successful experimental results, this study may open up new possibilities for future synthetic routes for manufacturing flexible energy storage devices employing environmental waste.

## 2. Results and Discussion

### 2.1. Fabrication of Positive and Negative Electrodes

**Figure 1:**
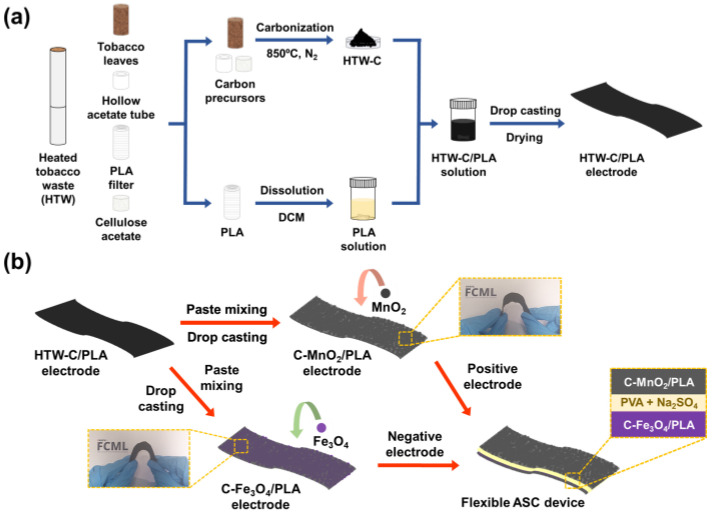
Schematic illustration for (**a**) full recycling of heated tobacco waste into flexible substrates and (**b**) the assembled flexible all-solid-state asymmetric supercapacitor (FASC) device based on C-MnO_2_/PLA as the positive electrode and C-Fe_3_O_4_/PLA as the negative electrode in this study.

The preparation strategy of the flexible electrodes for the practical all-solid-state asymmetric supercapacitor devices has been divided into two parts: (1) full recycling of heated tobacco and employing as active materials and flexible substrates for high-performance supercapacitor electrodes and (2) successful assembly and testing of supercapacitor device with high flexibility and maintaining capacitance under harsh bending conditions. Figure 1 is illustrating the full recycling method of heated tobacco waste into an all-solid-state supercapacitor device. In detail, heated tobacco wastes have been divided into tobacco leaves, hollow acetate tubes, PLA filters, and cellulose acetates. Among them, three wastes except PLA filters are employed as the carbon precursors, resulting in the heated tobacco waste-derived carbon (HTW-C) materials after carbonization. On the other hand, PLA filters can be well-dissolved into the DCM (dichloromethane) solution, resulting in a gel-like PLA solution. In detail, PLA is an aliphatic polyester, which is insoluble in polar solvents like water and alcohols, but is soluble in organic solvent [40,41]. Moreover, DCM is a fast-drying organic solvent; therefore, PLA-based flexible substrates are easily prepared by facile drop-casting and drying process of DCM dissolved PLA solution [42,43,44].

**Figure 2:**
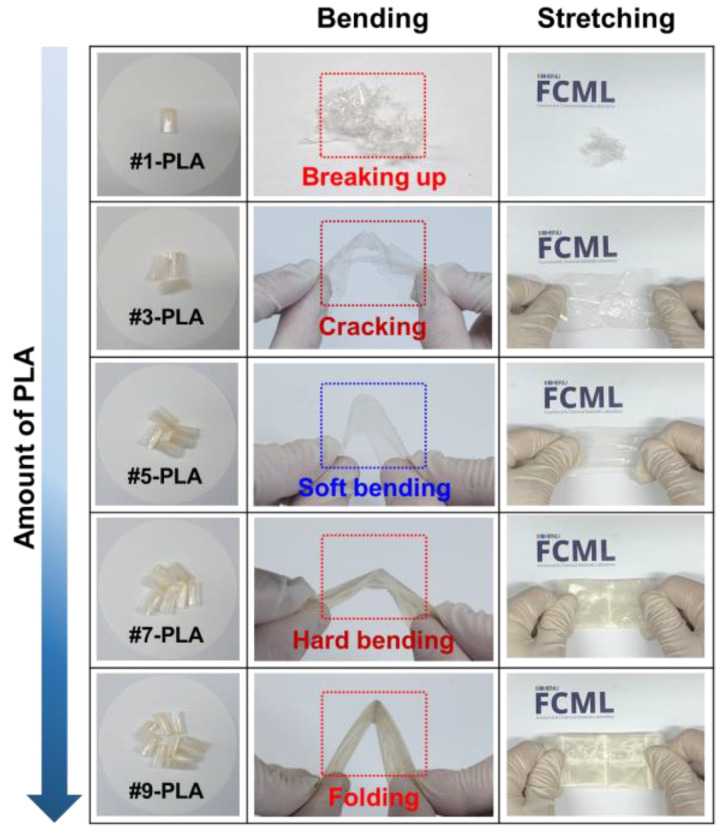
Digital photographs of bending and stretching tests for PLA substrates according to the added number of PLA filters.

Furthermore, the as-prepared solution is drop-casted onto the hot plate and the resulting HTW-C/PLA electrode is simply peeled off. It is noticeable that the flexibility of the HTW-C/PLA electrode has been manipulated by controlling the added number of PLA filters into the DCM solution. To optimize flexibility, the number of PLA filters dissolved in DCM (30 mL) has been controlled to 1, 3, 5, 7, and 9. After sonication and stirring, the fully dissolved PLA solutions are drop-casted onto a flat hot plate, dried, and peeled off to obtain PLA substrates, which are referred to as #1, 3, 5, 7, and 9-PLA substrates in this study. The bending and stretching tests are performed to determine the flexibility of the substrates, as shown in Figure 2. For #1-PLA substrate, it is extremely difficult to peel off and is torn into pieces. For #3-PLA substrate, a very thin flexible substrate is formed; however, after the bending and stretching tests, cracking problems occur. The substrate formed by the five PLA filters exhibits soft bending properties without cracking. The #5-PLA substrate also withstands the stretching tests. The #7-PLA substrate exhibits stiffness and starts losing flexibility. Eventually, the #9-PLA substrate is almost hardened, and folding occurs instead of bending. Based on the results of the bending and stretching tests, five PLA filters have been determined to be optimal for fabricating flexible substrates. Additionally, HTW-C materials have been mixed with each PLA solution, drop-casted, and peeled off to fabricate the HTW-C/PLA electrode. Thermogravimetric analysis (TGA) has been conducted to further study the properties of the PLA filters (Figure S1). All HTW-C/PLA electrodes exhibit mass losses between 300 and 400 °C. This mass loss originates from the decomposition of aliphatic polyester groups in the PLA filters [45,46,47]. The loss of weight increases as the number of PLA filters increases. This result suggests that HTW-C materials are thermally stable and only PLA filters affect the changes in weight.

The electrical conductivities of carbon-based electrodes can be optimized by adding metal oxides, resulting in an increase in capacitance [48,49,50]. In each HTW-C/PLA solution, MnO_2_ and Fe_3_O_4_ have been added, mixed, and drop-casted. The electrical conductivities of the C-MnO_2_/PLA and C-Fe_3_O_4_/PLA electrodes are measured as a function of the MnO_2_ and Fe_3_O_4_ materials added, respectively, and the results are plotted in Figure S2. For the C-MnO_2_/PLA electrode, the highest electrical conductivity is 378 μS cm^−1^ when 0.4 g of MnO_2_ materials are added. The C-Fe_3_O_4_/PLA electrode exhibits the highest electrical conductivity of 660 μS cm^−1^ by adding 0.3 g of Fe_3_O_4_ materials. It is noticeable that the additions of metal oxides firstly enhances the electrical conductivities of C-MnO_2_/PLA and C-Fe_3_O_4_/PLA electrodes, which may attribute to the substitution and filling effects of metal oxides onto the non-conductive PLA compositions. However, electrical conductivities of both C-MnO_2_/PLA and C-Fe_3_O_4_/PLA electrodes have decreased after passing 0.4 and 0.3 g, respectively. Such a phenomenon is ascribed to the naturally low electrical conductivities of metal oxides compared with that of carbon materials [51,52]. Taken together, electrical conductivities of electrodes are enhanced with the increased amount of metal oxides overcoming the low conduction nature of PLA, but after surpassing certain amounts, electrical conductivities are diminished due to the negative effect on the highly conductive carbon materials. Accordingly, the optimized adding amounts of MnO_2_ and Fe_3_O_4_ to the HTW-C/PLA solution are determined as 0.4 and 0.3 g, which maximizes the electrical conductivity while achieving the pseudocapacitance originating from the metal oxides.

**Figure 3:**
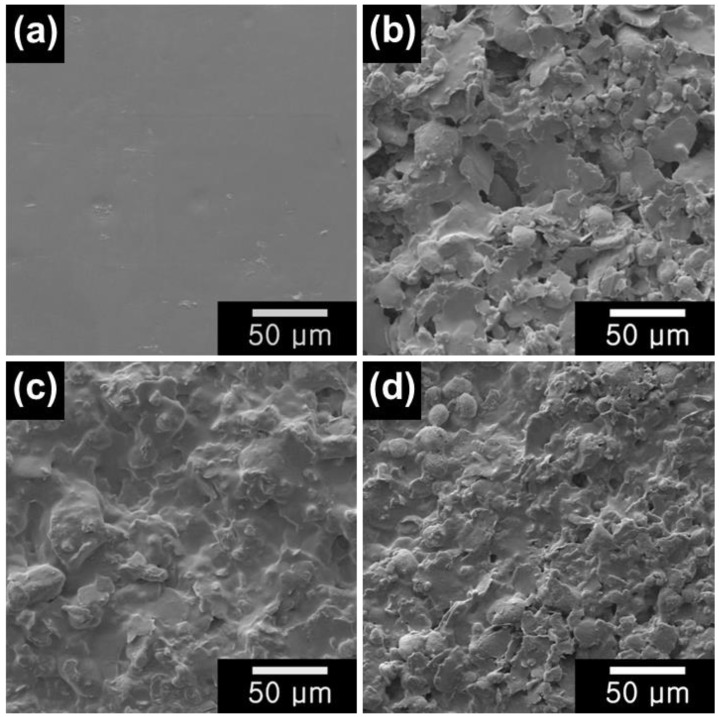
SEM images of (**a**) PLA substrate, (**b**) HTW-C/PLA, (**c**) C-MnO_2_/PLA, and (**d**) C-Fe_3_O_4_/PLA.

Scanning electron microscopy (SEM) analysis has been conducted to observe the morphological structures of a series of PLA-based materials (Figure 3). Without any addition of HTW-C and metal oxides, only the PLA substrate manifests a smooth surface (Figure 3a). The surface roughness increases with the addition of HTW-C and metal oxides, indicating the successful incorporation of materials into the PLA substrate (Figure 3b–d). Furthermore, energy-dispersive X-ray spectroscopy (EDS) analysis has been carried out to determine the elemental compositions, as listed in Table 1. Also, corresponding EDS spectra are shown in Figure S3. Only C and O elements are detected from the PLA substrate and HTW-C/PLA, which is in accordance with their inherent chemical compositions [53]. With the addition of HTW-C materials, amounts of the C element have been increased for the HTW-C/PLA, indicating the successful incorporation of carbons into PLA substrate. On the other hand, Mn and Fe elements are detected from the C-MnO_2_/PLA and C-Fe_3_O_4_/PLA electrodes, which further demonstrate the successful composition of MnO_2_ and Fe_3_O_4_ into the HTW-C/PLA via a simple paste-mixing method.

**Figure 4:**
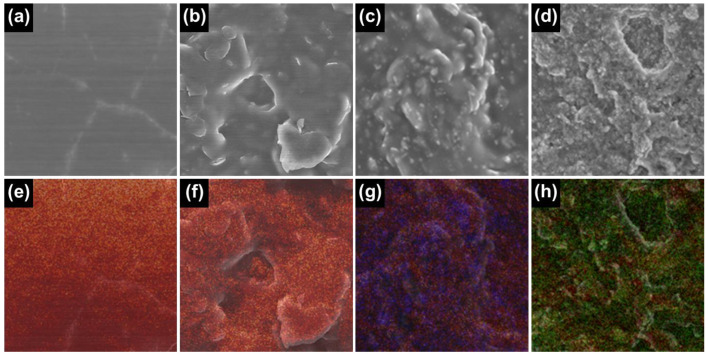
SEM and overlaid elemental mapping images of (**a**,**e**) PLA substrate, (**b**,**f**) HTW-C/PLA, (**c**,**g**) C-MnO_2_/PLA, and (**d**,**h**) C-Fe_3_O_4_/PLA, respectively [detected elements: C (red), O (yellow), Mn (blue) and Fe (green)].

To visualize the elemental compositions, elemental mapping analysis has further been carried out for a series of PLA-based materials (Figure 4). Only C (red) and O (yellow) elements have been observed for PLA substrate and HTW-C/PLA, which is in accordance with EDS data. For C-MnO_2_/PLA and C-Fe_3_O_4_/PLA, Mn (blue) and Fe (green) elements have been successfully detected. Also, Mn and Fe elements have thoroughly been detected in the entire region, indicating that the simple paste mixing method has homogenously mixed the HTW-C, PLA, MnO_2_, and Fe_3_O_4_.

**Figure 5:**
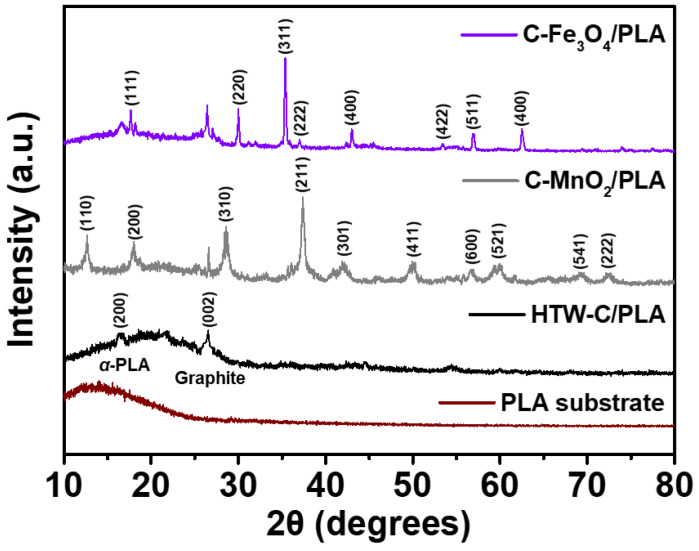
XRD spectra of PLA substrate, HTW-C/PLA, C-MnO_2_/PLA, and C-Fe_3_O_4_/PLA.

X-ray diffraction (XRD) patterns have been obtained to identify the crystal structures of the PLA substrate, HTW-C/PLA, C-MnO_2_/PLA, and C-Fe_3_O_4_/PLA (Figure 5). Firstly, the PLA substrate shows a broad peak between 10–26°, indicating the non-crystalline or amorphous characteristics [54,55]. However, the diffraction peaks of the HTW-C/PLA appear at 16.4 and 26°, corresponding to the (200) plane of *α* crystal PLA and (002) plane of graphitic carbon, respectively [56,57,58]. In detail, amorphous PLA substrate is dissolved by DCM and crystallinity (*α* crystal) is formed during the drop-casting and drying process [59]. In this regard, HTW-C/PLA manifest two characteristic peaks at 16.4 and 26°, indicating the successful incorporation of HTW-C and PLA substrate [60]. Moreover, C-MnO_2_/PLA and C-Fe_3_O_4_/PLA clearly manifest the characteristic peaks of MnO_2_ and Fe_3_O_4_. Specifically, diffraction peaks for C-MnO_2_/PLA are detected at 12.5, 18.4, 28.5, 36.8, 42.2, 50.0, 56.6, 60.0, 69.1, and 72.3°, corresponding to the (110), (200), (310), (211), (301), (411), (600), (521), (541), and (222) crystalline planes of tetragonal *α*-MnO_2_ (JCPDS 44-0141), respectively [61,62]. Also, diffraction peaks for C-Fe_3_O_4_/PLA are detected at 18.2, 30.1, 35.6, 36.9, 43.2, 53.1, 56.8, and 62.5°, which are indicating the (111), (220), (311), (222), (400), (422), (511), and (440) magnetic crystal reflections of Fe_3_O_4_ (JCPDS 75-0033), respectively [63,64]. Accordingly, XRD patterns of materials confirm the successful incorporation of MnO_2_ and Fe_3_O_4_ into the HTW-C/PLA electrodes.

### 2.2. Electrochemical Properties of C-MnO_2_/PLA and C-Fe_3_O_4_/PLA in a Three Electrode System

**Figure 6:**
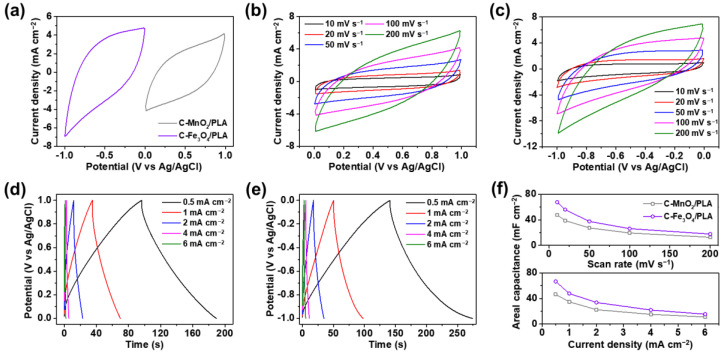
(**a**) Cyclic voltammetry (CV) curves of C-MnO_2_/PLA and C-Fe_3_O_4_/PLA at a scan rate of 100 mV s^−1^. CV curves of (**b**) C-MnO_2_/PLA and (**c**) C-Fe_3_O_4_/PLA at various scan rates. Galvanostatic charge-discharge (GCD) curves of (**d**) C-MnO_2_/PLA and (**e**) C-Fe_3_O_4_/PLA at different current densities. (**f**) Areal capacitances of C-MnO_2_/PLA and C-Fe_3_O_4_/PLA calculated from CV (top) and GCD (bottom) curves.

Cyclic voltammetry (CV) and galvanostatic charge-discharge (GCD) curves of C-MnO_2_/PLA and C-Fe_3_O_4_/PLA have been measured in a three-electrode system to examine the suitability of the electrodes as energy storage devices. The three-electrode system consists of the as-prepared working electrode, a platinum wire as the counter electrode, and Ag/AgCl as the reference electrode in a 1 M Na_2_SO_4_ aqueous electrolyte solution at 25 °C. Figure 6a shows the CV curves for C-MnO_2_/PLA as the positive electrode and C-Fe_3_O_4_/PLA as the negative electrode at a scan rate of 100 mV s^−1^. The operating potential range of C-MnO_2_/PLA has been measured from 0 to 1.0 V, while the range of C-Fe_3_O_4_/PLA has been determined from −1.0 to 0 V. Electrodes with different operating voltage windows in same electrolyte can extend the voltage window and allow them to be used as the positive and negative electrodes for supercapacitors [65,66,67].

Figure 6b shows the CV curves of C-MnO_2_/PLA in the operating potential range of 0–1.0 V at different scan rates ranging from 10 to 200 mV s^−1^. The specific capacitances of C-MnO_2_/PLA calculated from the CV curves are 47.8, 38.9, 27.5, 19.5, and 12.8 mF cm^−2^ at scan rates of 10, 20, 50, 100, and 200 mV s^−1^, respectively. As the scan rate increases, the charge-transfer rate in the electrode becomes faster than the ion-diffusion rate at the interface between the electrode and electrolyte, resulting in a deviation from the original shape and a slight decrement in the specific capacitance and rate capability of electrodes [68,69,70]. Also, the absence of the current collector and employing the non-conductive PLA may attribute to the decreased capacitances of electrodes at high scan rates by degrading the redox reaction of metal oxides [71]. Figure 6c presents the CV curves of C-Fe_3_O_4_/PLA in the operating potential range of −1.0–0 V at various scan rates. The specific capacitances of C-Fe_3_O_4_/PLA evaluated from the CV curves are 68.0, 56.1, 37.6, 26.3, and 16.1 mF cm^−2^ at scan rates of 10, 20, 50, 100, and 200 mV s^−1^, respectively. With increasing scan rates, similar phenomenon in the decrement of specific capacitance is observed like C-MnO_2_/PLA. For the quantitative analysis and comparison purpose, CV curves of the HTW-C/PLA are determined in the potential range of −1.0–0 V at different scan rates from 10 to 200 mV s^−1^ (Figure S4). From the CV curves, specific capacitances of HTW-C/PLA electrodes are calculated as 30.5, 20.8, 12.2, 7.8, and 5.3 mF cm^−2^ at 10, 20, 50, 100, and 200 mV s^−1^, respectively. Noticeably, HTW-C/PLA manifests ca. 1.6- and 2.2-folds lower specific capacitances compared with that of C-MnO_2_/PLA (47.8 mF cm^−2^) and C-Fe_3_O_4_/PLA (68.0 mF cm^−2^) at a scan rate of 10 mV s^−1^, respectively. Such enhanced specific capacitances of C-MnO_2_/PLA and C-Fe_3_O_4_/PLA owe to the addition of pseudocapacitive characteristics to the HTW-C/PLA, which only possess EDLC characteristics originating from the carbonaceous HTW-C material [72].

The GCD curves of C-MnO_2_/PLA and C-Fe_3_O_4_/PLA have been obtained to study their electrochemical properties further. Figure 6d shows the GCD curves of C-MnO_2_/PLA in the potential range of 0–1.0 V at different current densities ranging from 0.5 to 6 mA cm^−2^. The GCD curves show excellent capacitive behavior without apparent IR drops. The areal capacitances of C-MnO_2_/PLA calculated from the GCD curves are 46.4, 34.8, 22.5, 11.0, and 5.7 mF cm^−2^ at current densities of 0.5, 1, 2, 4, and 6 mA cm^−2^, respectively. Notably, the charging and discharging times are almost balanced. In the case of 1 mA cm^−2^, the charging and discharging times are 35.0 and 34.7 s, respectively. This result suggests an outstanding coulombic efficiency, indicating excellent capacitive performance and reversibility [73,74,75]. The GCD curves of C-Fe_3_O_4_/PLA are measured in the potential range of −1.0–0 V at different current densities, as shown in Figure 6e. The areal capacitances of C-Fe_3_O_4_/PLA computed from the GCD curves are 66.7, 47.9, 33.8, 22.0, and 15.4 mF cm^−2^ at current densities of 0.5, 1, 2, 4, and 6 mA cm^−2^, respectively. The charging and discharging times at a current density of 1 mA cm^−2^ are 140.8 and 133.3 s, respectively, implying excellent coulombic efficiency. The evaluated specific capacitances of C-MnO_2_/PLA and C-Fe_3_O_4_/PLA from the CV and GCD curves are shown in the bottom part of Figure 6f.

Furthermore, the electrochemical impedance spectroscopy (EIS) of C-MnO_2_/PLA and C-Fe_3_O_4_/PLA has been analyzed to compare the electrochemical properties of electrodes (Figure S5). The values of equivalent series resistance ($R_{\mathrm{ESR}}$) for C-MnO_2_/PLA and C-Fe_3_O_4_/PLA are measured as 9.03 and 4.94 Ω, respectively. Such EIS results are in accordance with the electrical conductivities of C-MnO_2_/PLA and C-Fe_3_O_4_/PLA electrodes. As discussed previously, the added amounts of MnO_2_ and Fe_3_O_4_ to HTW-C/PLA are determined to be 0.4 and 0.3 g, according to the electrical conductivity optimization. With the increased amount of metal oxide, C-MnO_2_/PLA shows lower electrical conductivity compared to C-Fe_3_O_4_/PLA electrodes. In this regard, C-MnO_2_/PLA manifests slightly higher $R_{\mathrm{ESR}}$ than C-Fe_3_O_4_/PLA; however, both electrodes possess sufficiently low resistance for the supercapacitor application.

### 2.3. Electrochemical Analysis of Assembled Flexible Asymmetric Supercapacitor (FASC) Device

A flexible all-solid-state asymmetric supercapacitor device has been assembled with the as-prepared C-MnO_2_/PLA as the positive electrode, C-Fe_3_O_4_/PLA as the negative electrode, and PVA as the solid electrolyte with the addition of Na_2_SO_4_. In general, charge balance needs to be calculated to prepare an effective all-solid-state asymmetric supercapacitor device [76]. For the electrode configuration, the charge balance between the positive and negative electrodes is a significant factor for the electrochemical performance of a supercapacitor to minimize the loss of capacitance [77,78,79]. The charge storage can be calculated using the following equation [80]:

(1) Q=C×ΔV×S

where Q is the electrode charge, C is the capacitance, ΔV is the operating voltage window, and S is the surface area. To satisfy the balance between the positive electrode charge $Q_{+}$ and the negative electrode charge $Q_{-}$, the areal ratio $S_{+}$/$S_{-}$ can be evaluated using the following equation:

(2) $$\frac{S_{+}}{S_{-}}=\frac{C_{-}\times \Delta V_{-}}{C_{+}\times \Delta V_{+}}$$

The calculated areal ratios from CV results of C-MnO_2_/PLA and C-Fe_3_O_4_/PLA in the three electrode system are 1.42, 1.44, 1.37, 1.35, and 1.39 at scan rates of 10, 20, 50, 100, and 200 mV s^−1^, respectively. From GCD results, the ratios are 1.44, 1.38, 1.45, 1.44, and 1.39 at current densities of 0.5, 1, 2, 4, and 6 mA cm^−2^, respectively. Based on the areal ratios obtained from CV and GCD at various scan rates and current densities, the optimal areal ratios of C-MnO_2_/PLA and C-Fe_3_O_4_/PLA are 1.41 for the FASC device. Accordingly, the FASC device has successfully been prepared with the assembly of C-MnO_2_/PLA and C-Fe_3_O_4_/PLA electrodes by accounting the calculated charge balance ratio of metal oxides.

**Figure 7:**
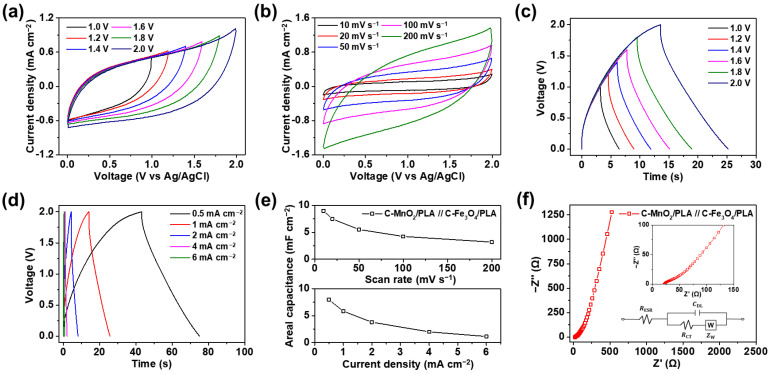
CV curves of the as-prepared C-MnO_2_/PLA//C-Fe_3_O_4_/PLA FASC device in this study with (**a**) different voltage ranges and (**b**) scan rates. GCD curves of the FASC device with (**c**) different voltage windows and (**d**) current densities. (**e**) Areal capacitances of the FASC device calculated from CV (top) and GCD (bottom) curves in the operating voltage range of 2.0 V. (**f**) Electrochemical impedance spectroscopy (EIS) analysis of the FASC device.

The practical electrochemical performance of the as-prepared C-MnO_2_/PLA//C-Fe_3_O_4_/PLA FASC device has been investigated to analyze the suitability as a flexible energy storage device. Figure 7a presents the CV curves of the FASC device at a scan rate of 100 mV s^−1^ in the PVA/Na_2_SO_4_ gel electrolyte in the potential windows of 1.0, 1.2, 1.4, 1.6, 1.8, and 2.0 V. As shown in the figure, the CV curves of the FASC device based on C-MnO_2_/PLA and C-Fe_3_O_4_/PLA are operated up to 2.0 V owing to the large work function difference between the positive and negative electrodes. Figure 7b shows the CV curves of the FASC device at scan rates of 10, 20, 50, 100, and 200 mV s^−1^ in a voltage window of 2.0 V. The capacitive behavior is maintained at all scan rates. The specific capacitances of the FASC device, calculated from the CV curves, are 9.00, 7.49, 5.53, 4.24, and 3.19 mF cm^−2^ at scan rates of 10, 20, 50, 100, and 200 mV s^−1^, respectively.

Furthermore, the GCD curves of the FASC device at a current density of 1 mA cm^−2^ in the operating voltage windows from 1.0 to 2.0 V are measured to elucidate the electrochemical behavior of the FASC device (Figure 7c). The slight slope change in the GCD curves is attributed to the redox reactions caused by the metal oxides [81]. As the operating voltage window increases, the specific capacitance also increases, owing to the pseudocapacitive reaction. The specific capacitances evaluated from the GCD curves at a current density of 1 mA cm^−2^ are 3.21, 3.68, 4.11, 4.60, 5.22, and 5.80 mF cm^−2^ in the operating voltage windows of 1.0, 1.2, 1.4, 1.6, 1.8, and 2.0 V, respectively. The coulombic efficiency calculated from GCD curves are 99.5, 96.7, 93.7, 94.9, 98.4, and 86.0 % in voltage windows of 1.0, 1.2, 1.4, 1.6, 1.8, and 2.0 V, respectively, which suggests outstanding reversible properties. Figure 7d shows the GCD curves of the FASC device in the voltage range windows of 2.0 V at different current densities. The specific capacitances calculated from GCD curves are 7.97, 5.80, 3.80, 2.00, and 1.14 mF cm^−2^ at current densities of 0.5, 1, 2, 4, and 6 mA cm^−2^, respectively. The specific capacitances of the FASC device as functions of the scan rates and current densities in the operating voltage range of 2.0 V are plotted on the top and bottom sides of Figure 7e, respectively.

Finally, electrochemical impedance spectroscopy (EIS) has been further analyzed to investigate the ion-transport capabilities of the as-prepared FASC device (Figure 7f). Moreover, a corresponding equivalent circuit diagram of the device is inserted as inset, including equivalent series resistance ($R_{\mathrm{ESR}}$), charge-transfer resistance ($R_{\mathrm{CT}}$), Warburg impedance ($Z_{W}$), and double-layer capacitance ($C_{\mathrm{DL}}$) [82]. In specific, $R_{\mathrm{ESR}}$, meaning the combined resistance of ionic resistance of the electrolyte and surface resistance of active materials, is measured as 21.5 Ω in high-frequency region. Also, $R_{\mathrm{CT}}$, corresponding to the resistance caused by the ionic reaction between the electrolyte and electrode, is determined as 37.8 Ω. Furthermore, a straight line with high linear slope investigated from the low-frequency region corresponds to the Warburg diffusion, suggesting the excellent capacitive behavior of the device [83]. To sum up, low $R_{\mathrm{ESR}}$ and $R_{\mathrm{CT}}$ values with a high linear slope in the low frequency region indicates the excellent ion transport capabilities of the as-prepared FASC device.

**Figure 8:**
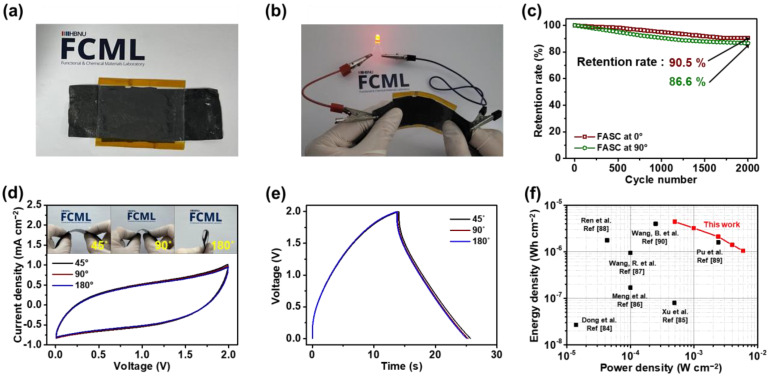
Digital photograph of (**a**) C-MnO_2_/PLA//C-Fe_3_O_4_/PLA FASC device, and (**b**) FASC device lightning LED. (**c**) Long-term cycling test of the FASC device at bending angles of 0 and 90° with the application of current density of 4 mA cm^−2^. (**d**) CV and (**e**) GCD curves of the FASC device at bending angles of 45, 90, and 180°. (**f**) Ragone plot of the FASC device compared to various flexible supercapacitor devices.

Figure 8a shows a digital photograph of the as-prepared C-MnO_2_/PLA//C-Fe_3_O_4_/PLA FASC device. For the practical assembly, a gel electrolyte is prepared by mixing 1 M Na_2_SO_4_ with a PVA (10.0 wt%) solution and inserting it between as-synthesized C-MnO_2_/PLA and C-Fe_3_O_4_/PLA electrodes. Also, transparent polyimide film is applied on the outer surface of FASC device for the enhancement of contact between the solid electrolyte and electrodes. Noticeably, the as-prepared FASC device is able to illuminate the red LED (1.8 V), indicating the wide operating window originated from the successful incorporation of MnO_2_ and Fe_3_O_4_ to the HTW-C/PLA matrix (Figure 8b). In addition, a cyclability test on FASC devices has been carried out to ensure the stability of the device at a current density of 4 mA cm^−2^ for 2000 cycles (Figure 8c). Also, the stability test is measured at the bending angle of 0 and 90°. The specific capacitances of the FASC devices at the bending angle of 0 and 90° are similarly determined as ca. 90.5 and 86.6 % of its initial value, indicating the excellent cycling and stability at harsh bending conditions. Furthermore, CV and GCD curves of FASC device at different bending angles of 45, 90, and 180° have been measured, as shown in Figure 8d,e. Notably, resulting CV and GCD curves maintain the stable shape even at severe angles, which verifies the excellent stability of the FASC device. Lastly, energy density and power density of the as-prepared FASC device are compared with other flexible devices (Figure 8f) [84,85,86,87,88,89,90]. Particularly, FASC manifests a maximum energy density of 4.43 μWh cm^−2^ at a current density of 0.5 mA cm^−2^. With the increasing current density, the energy density of the FASC device has decreased, while the power density of the FASC device manifests increasing trends and maximizes to 5.94 mW cm^−2^ at a current density of 6 mA cm^−2^. Compared to other flexible supercapacitors, the as-prepared FASC device displays sufficiently high energy density with high power density, demonstrating the excellent performance of the FASC device. This outstanding supercapacitor performance is ascribed to the high capacitance of C-MnO_2_/PLA and C-Fe_3_O_4_/PLA electrodes and the extended voltage window of the FASC device. Hence, the as-prepared C-MnO_2_/PLA//C-Fe_3_O_4_/PLA FASC device with high performance shows the possibility for future eco-friendly energy storage device.

## 3. Conclusions

In summary, heated tobacco waste has been fully recycled into a C-MnO_2_/PLA//C-Fe_3_O_4_/PLA FASC device via a facile PLA gelation template method. Heated tobacco has more leftovers than conventional tobacco due to differences in smoking mechanisms. Tobacco leaves and filter tubes from heated tobacco waste have been employed as carbon precursors, and PLA filters have been used as flexible binding substrates. Additionally, introducing metal oxides (MnO_2_ and Fe_3_O_4_) to heated tobacco waste-derived carbon materials extends the operating voltage range and improves the electrical conductivity and electrochemical performance. Flexible C-MnO_2_/PLA and C-Fe_3_O_4_/PLA electrodes have been successfully fabricated via a series of PLA gelation templates, simple paste mixing, and drop-casting methods. As-synthesized C-MnO_2_/PLA and C-Fe_3_O_4_/PLA electrodes exhibit remarkable specific capacitance of 34.8 and 47.9 mF cm^−2^ at 1 mA cm^−2^, respectively. Furthermore, the FASC device is assembled using two electrodes: C-MnO_2_/PLA as the positive electrode and C-Fe_3_O_4_/PLA as the negative electrode. The as-prepared FASC device displays an excellent capacitance of 5.80 mF cm^−2^ at 1 mA cm^−2^ and is able to illuminate the red LED. The FASC device exhibits stable electrochemical performance under various harsh bending and stretching conditions of 45, 90, and 180°. In this regard, we expect that it could pave the way for the development of future flexible and wearable electronic devices employing environmental waste.

## 4. Materials and Methods

### 4.1. Materials

Heated tobacco waste from Heats Silver (Philip Morris Int., New York, NY, USA) was collected after being smoked. Dichloromethane (DCM, 99.5%), manganese dioxide powder (MnO_2_), iron(II,III) oxide black powder (Fe_3_O_4_), and sodium sulfate (Na_2_SO_4_) were purchased from Samchun Chemical Company (Seoul, Republic of Korea). Poly(vinyl alcohol) (PVA, *M*_w_ 89,000–98,000) was purchased from Sigma-Aldrich Co. (Burlington, MA, USA).

### 4.2. Preparation of Heated Tobacco Waste-Derived Carbon/PLA (HTW-C/PLA) Electrodes

Heated tobacco waste was collected and divided into separate parts of tobacco leaves, hollow acetate tubes, cellulose acetates, and poly(lactic acid) filters. Carbon precursors from the heated tobacco waste were collected and carbonized under N_2_ atmosphere at 850 °C for 2 h at a heating rate of 5 °C min^−1^ to obtain heated tobacco waste-derived carbon (HTW-C) materials. Moreover, PLA filters were dropped into DCM (30 mL) to form a PLA solution and were treated under 15 min of sonication until complete dissolution, then stirred for 30 min at room temperature. The number of PLA filters was controlled to 1, 3, 5, 7, and 9 to examine the conditions of the PLA substrate after drop-casting. The mass of each PLA filter was measured as ca. 0.225 g. While stirring, HTW-C materials (1.0 g) were augmented into the PLA solution, and the resulting HTW-C/PLA solution was stirred for an additional 6 h. HTW-C/PLA electrodes were fabricated by drop-casting a HTW-C/PLA solution onto a flat hot plate while maintaining the temperature of the plate at 50 °C for 15 min. After lowering the temperature of the plate to RT, several ice cubes were laid on the dried solution to peel off the resulting HTW-C/PLA electrode more easily. A detailed schematic for the fabrication of HTW-C/PLA was shown in Figure 1a.

### 4.3. Fabrication of C-MnO_2_/PLA as a Positive Electrode and C-Fe_3_O_4_/PLA as a Negative Electrode

C-MnO_2_/PLA was used as a positive electrode. MnO_2_ nanoparticles were dispersed in 30 mL of the HTW-C/PLA solution and stirred for an additional 3 h. As a negative electrode, Fe_3_O_4_ was mixed to HTW-C/PLA. The Fe_3_O_4_ black powder was dispersed in 30 mL of HTW-C/PLA solution. Both MnO_2_- and Fe_3_O_4_-added HTW-C/PLA solutions were shuffled using a paste mixer (PDM-300, DAE WHA TECH, Gyeonggi-do, Republic of Korea) at a rotation speed of 850 rpm and revolution of 750 rpm for 2 h. The added amounts of MnO_2_ and Fe_3_O_4_ for each electrode were controlled between 0.1 and 0.6 g to identify the optimal mass for electrical conductivities. The resulting MnO_2_- and Fe_3_O_4_-added HTW-C/PLA solutions were employed to fabricate C-MnO_2_/PLA and C-Fe_3_O_4_/PLA electrodes, respectively, using the drop-casting method described in Section 4.2. The as-fabricated C-MnO_2_/PLA and C-Fe_3_O_4_/PLA electrodes were cut into rectangular shapes to construct flexible asymmetric all-solid-state supercapacitor devices.

### 4.4. Assembly of the Flexible All-Solid-State Asymmetric Supercapacitor (FASC) Device

Prior to the preparation of a flexible all-solid-state asymmetric supercapacitor, the PVA/Na_2_SO_4_ gel electrolyte was fabricated. PVA powder (4.0 g) was dispersed in DI water (40 mL) via magnetic stirring for 10 h at 85 °C until complete dissolution. Subsequently, 5.77 g of Na_2_SO_4_ was added and vigorously stirred for 30 min. The solution was then left at room temperature to form a gel electrolyte, which was stored. The assembly of the practical FASC device was constructed using C-MnO_2_/PLA and C-Fe_3_O_4_/PLA as positive and negative electrodes, respectively. The calculated ideal areal ratios of C-MnO_2_/PLA and C-Fe_3_O_4_/PLA were 1.41:1 to match the charge balance between the two electrodes. To fabricate the FASC device, a minimal amount of PVA/Na_2_SO_4_ gel electrolyte was coated on the surfaces of the C-MnO_2_/PLA and C-Fe_3_O_4_/PLA electrodes and left in a fume hood for 10 h to evaporate water. Then, C-MnO_2_/PLA and C-Fe_3_O_4_/PLA electrodes were physically assembled by pressing the gel-coated sides together and transparent polyimide films were coated onto the outer surface for enhancing the contacts of electrodes and electrolytes. Finally, a flexible all-solid-state asymmetric supercapacitor (C-MnO_2_/PLA//C-Fe_3_O_4_/PLA) device was successfully prepared for the electrochemical application and various analysis.

### 4.5. Characterization

The morphologies of the prepared electrodes were investigated using field-emission scanning electron microscopy (FE-SEM; S-4800, Hitachi, Tokyo, Japan). The elemental compositions of C, O, Mn, and Fe in the fabricated electrodes were obtained using an energy-dispersive X-ray spectroscopy (EDS) system (EX-250, HORIBA Ltd., Kyoto, Japan) installed in the FE-SEM instrument. Thermograms of the HTW-C/PLA electrodes were obtained using a thermogravimetric analyzer (TGA2, Mettler Toledo GmbH, Columbus, OH, USA). The electrical conductivities of the C-MnO_2_/PLA and C-Fe_3_O_4_/PLA electrodes were obtained using a two-point probe system (MCP-HT450, Mitsubishi, Tokyo, Japan) after being cast in the pellet form. The crystal structures of the prepared electrodes were investigated by X-ray diffraction (XRD, D8 Advance, Bruker Co., Billerica, MA, USA) in the 2θ range of 10–80° at 10° min^−1^.

### 4.6. Electrochemical Measurement

Cyclic voltammetry (CV), galvanostatic charge-discharge (GCD), and electrochemical impedance spectroscopy (EIS) measurements were carried out on an electrochemical workstation (ZIVE SP1, WonATech Co., Seoul, Republic of Korea). The CVs and GCDs of the C-MnO_2_/PLA and C-Fe_3_O_4_/PLA electrodes were performed in a traditional three-electrode system consisting of an Ag/AgCl (saturated by 3 M NaCl) assembly and a platinum wire as the reference and counter electrodes, respectively. As an electrolyte, 1 M Na_2_SO_4_ aqueous solution was used for the system. The potential range of C-MnO_2_/PLA and C-Fe_3_O_4_/PLA electrodes were set to 0–1.0 V and −1.0–0 V, respectively. The CVs and GCDs were measured by varying the scan rate from 10 to 200 mV s^−1^ and the current density from 0.5 to 6 mA cm^−2^, respectively. The current density of the GCD was evaluated by dividing the surface area of the impregnated electrode from the current passing through the electrode [91]. The areal capacitances (mF cm^−2^) were calculated from the CV and GCD curves using the following equation [92,93]:

(3) $$C=\frac{1}{S\times \nu \times \Delta V}\;\int_{V_{0}}^{V_{0}+\Delta V}IdV\;\left(\mathrm{from}\;\mathrm{CV}\;\mathrm{curves}\right)$$

(4) $$\;C=\frac{I\times \Delta t}{S\times \Delta V}\;\left(\mathrm{from}\;\mathrm{GCD}\;\mathrm{curves}\right)$$

where C is the areal capacitance, ν is the scan rate, I is the discharging current, V is the voltage, ΔV is the operating voltage window, Δt is the discharging time, and S is the electrode surface area. The CVs and GCDs of the FASC device were measured under the same conditions as the three-electrode system, except for the operating voltage window of 2.0 V. The areal energy density (μWh cm^−2^) and power density (μW cm^−2^) of the device were then calculated by the subsequent equations [94,95]:

(5) $$E=\frac{C\times \Delta V^{2}}{2}\times \frac{1000}{3600}$$

(6) $$P=\frac{E}{\Delta t}\times 3600$$

where E is the areal energy density, and P is the areal power density.

Electrochemical impedance spectroscopy of the FASC device was recorded by applying an AC voltage of 10 mV in the frequency range of 10 mHz to 100 kHz.

## Figures and Tables

**Table 1:** Elemental compositions of various PLA-based materials synthesized in this study ^a^.

Sample	Element (Atomic %)			
	C	O	Mn	Fe
PLA substrate	65.02	34.98	-	-
HTW-C/PLA	77.32	22.68	-	-
C-MnO_2_/PLA	62.25	27.63	10.12	-
C-Fe_3_O_4_/PLA	60.78	23.72	-	15.34

^a^ Elemental composition of the samples was obtained by the EDS mode installed in FE-SEM (beam current: 10.0 μA, accelerating voltage: 10.0 kV).

## Data Availability

Data are contained within the article.

## Supplementary Materials

The following supporting information can be downloaded at: https://www.mdpi.com/article/10.3390/gels9020097/s1, Figure S1: TGA curves of HTW-C/PLA electrodes according to number of PLA filters. Figure S2: Electrical conductivities of C-MnO_2_/PLA and C-Fe_3_O_4_/PLA electrode according to the added amount of MnO_2_ and Fe_3_O_4_ materials, respectively. Figure S3: EDS spectra of (a) PLA substrate, (b) HTW-C/PLA, (c) C-MnO_2_/PLA, and (d) C-Fe_3_O_4_/PLA (detected elements: C, O, Mn and Fe). Figure S4: Cyclic voltammetry (CV) curves of HTW-C/PLA at various scan rates. Figure S5: Electrochemical impedance spectroscopy (EIS) analysis of C-MnO_2_/PLA and C-Fe_3_O_4_/PLA.
