# Supplementary material for: Fabrication of Flexible All-Solid-State Asymmetric Supercapacitor Device via Full Recycling of Heated Tobacco Waste Assisted by PLA Gelation Template Method
Source: Gels. 2023 Jan 23;9(2):97. doi: 10.3390/gels9020097 (PMC9956904; doi:10.3390/gels9020097)
Supplement: Supplementary file 1 [file gels-09-00097-s001.zip › gels-2167881-supplementary.pdf]

gels

Supplementary Materials

Fabrication of Flexible All-Solid-State Asymmetric Supercapacitor Device via Full Recycling of Heated Tobacco Waste Assisted by PLA Gelation Template Method

Suk Jekal [1,†], Min-Sang Kim [1,†], Dong-Hyun Kim [1], Jungchul Noh [2], Ha-Yeong Kim [1], Jiwon Kim [1], Hyeonseok Yi [3], Won-Chun Oh [4] and Chang-Min Yoon [1,*]

[1] Department of Chemical and Biological Engineering, Hanbat National University, Daejeon 34158, Republic of Korea; neofgreen@hanbat.ac.kr (S.J.); 20181304@edu.hanbat.ac.kr (M.-S.K.); 20181302@edu.hanbat.ac.kr (D.-H.K.); 20191363@edu.hanbat.ac.kr (H.-Y.K.); 20191360@edu.hanbat.ac.kr (J.K.)
[2] McKetta Department of Chemical Engineering and Texas Material Institute, The University of Texas at Austin, Austin, TX 78712, USA; jn0118@utexas.edu
[3] Institute for Materials Chemistry and Engineering, Kyushu University, Fukuoka 816-8580, Japan; yi@cm.kyushu-u.ac.jp.
[4] Department of Advanced Materials Science & Engineering, Hanseo University, Seosan-si 31962, Republic of Korea; wc_oh@hanseo.ac.kr.
* Correspondence: cmyoon4321@hanbat.ac.kr; Tel.: +82-42-821-1528; Fax: +82-42-821-1593
† These authors equally contributed to this work.

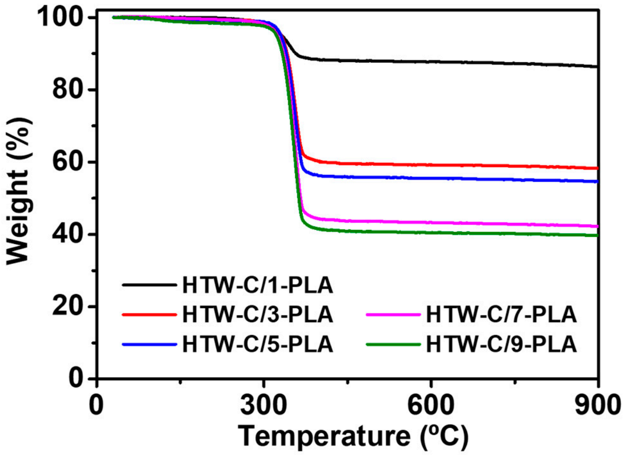

Figure S1. TGA curves of HTW-C/PLA electrodes according to number of PLA filters.

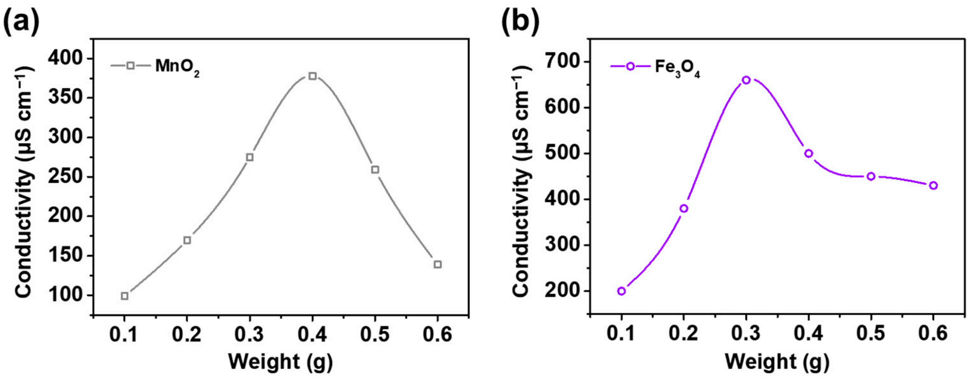

Figure S2. Electrical conductivities of C-MnO_2/PLA and C-Fe_3O_4/PLA electrode according to the added amount of MnO_2 and Fe_3O_4 materials, respectively.

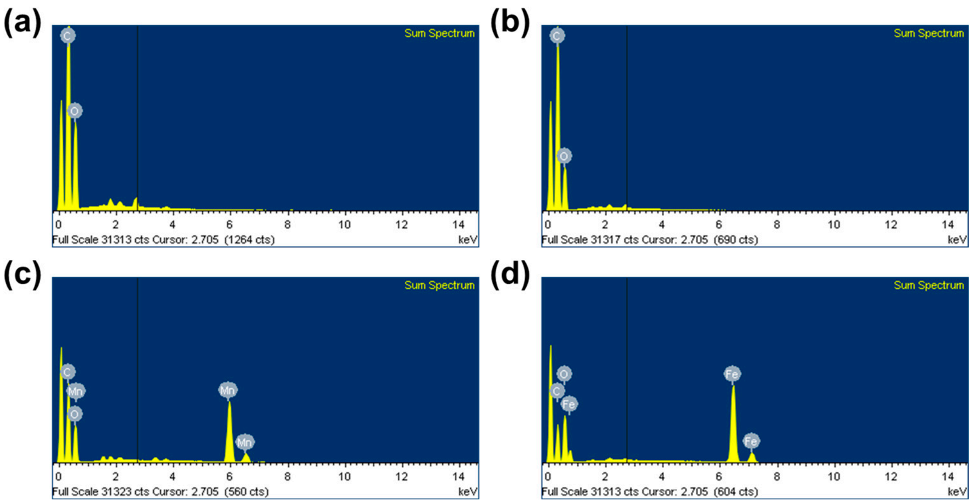

Figure S3. EDS spectra of (a) PLA substrate, (b) HTW-C/PLA, (c) C-MnO_2/PLA, and (d) C-Fe_3O_4/PLA (detected elements : C, O, Mn and Fe)

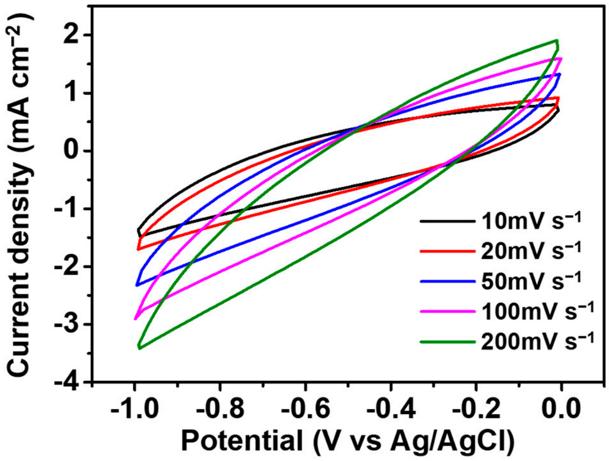

Figure S4. Cyclic voltammetry (CV) curves of HTW-C/PLA at various scan rates.

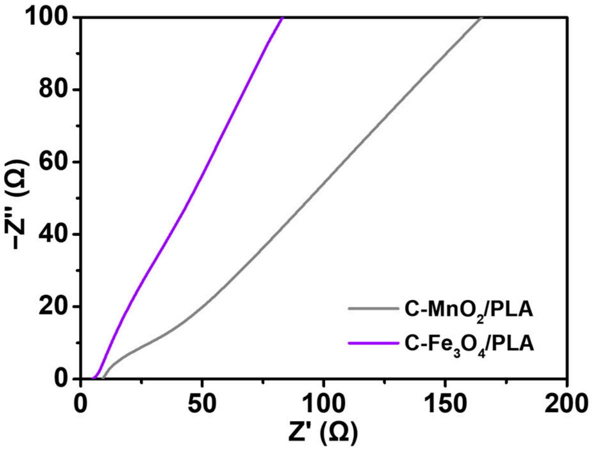

Figure S5. Electrochemical impedance spectroscopy (EIS) analysis of C-MnO_2/PLA and C-Fe_3O_4/PLA.
